# Efficacy Assessment of Phentolamine Accompanied by Lidocaine Subcutaneously under Ultrasound Guidance on Radial Artery Catheterization in Pediatric Patients

**DOI:** 10.1155/2022/6554993

**Published:** 2022-06-16

**Authors:** Erliang Kong, Lun Shu, Chang Yuan, Jianxin Wang, Feixiang Wu, Hongbin Yuan, Xudong Feng

**Affiliations:** ^1^Department of Anesthesiology, The 988th Hospital of Joint Logistic Support Force of Chinese People's Liberation Army, Zhengzhou, Henan 450042, China; ^2^Department of Anesthesiology, Changzheng Hospital, Second Affiliated Hospital of Naval Medical University, Shanghai 200003, China; ^3^Department of Anesthesiology, The First Affiliated Hospital of Zhengzhou University, Zhengzhou, Henan 450052, China; ^4^Department of Intensive Care Unit, Shanghai Eastern Hepatobiliary Surgery Hospital, Naval Medical University, Shanghai 200438, China

## Abstract

**Objective:**

Pediatric patients are facing greater difficulties in radial catheterization for anatomic variation and smaller diameter. This study is to investigate the efficacy of phentolamine accompanied by lidocaine subcutaneously under ultrasound guidance on radial catheterization in pediatric patients.

**Methods:**

66 pediatric patients were enrolled and randomly divided into saline group, phentolamine group, and phentolamine+lidocaine group. Baseline characteristics and surgical types were collected. Relevant solutions were subcutaneously injected, and catheterization was subsequently conducted under ultrasound guidance. Radial artery diameter and depth were measured, the success rate of catheterization and procedure time were calculated, and the complications were evaluated with ultrasonography.

**Results:**

No significant differences were observed in age, sex, weight, American Society of Anesthesiologists' classification, systolic blood pressure, diastolic blood pressure, heart rate, hemoglobin, and surgical types among three groups. Subcutaneously, the diameter in phentolamine and phentolamine+lidocaine groups increased significantly compared with the saline group. Moreover, the diameter also increased significantly after injection compared with that before injection both in the phentolamine and phentolamine+lidocaine groups. The first-attempt success rates were significantly higher while the procedure times of cannulation were shorter in the phentolamine and phentolamine+lidocaine groups than that in the saline group. Kaplan–Meier analysis showed that the overall procedure time was shorter in the phentolamine and phentolamine+lidocaine groups than the saline group. Overall complications and vasospasm incidence were lower in the phentolamine and phentolamine+lidocaine groups than the saline group.

**Conclusion:**

Phentolamine accompanied by lidocaine subcutaneous injection under ultrasound guidance improved the first-attempt success rate and reduced the complication of radial artery catheterization in pediatric patients.

## 1. Introduction

Percutaneous artery puncture technology is an important method for clinical interventional therapy and continuous blood pressure monitoring [1]. The radial artery is the most commonly used site for arterial catheterization to monitor invasive arterial pressure for its superficial location, larger inner diameter, less trauma, and quicker recovery compared with other peripheral arterial vessels [2, 3]. Large numbers of pediatric patients need surgical treatment every year, such as heart surgery, neurosurgery, or general surgery, and dynamic invasive blood pressure monitoring in such surgeries exerts a vital role in ensuring safety in perioperative period [4]. However, radial artery catheterizations are facing great difficulty for the disadvantages of low development, small inner diameter, and variation in pediatric patients. Studies have shown that the first success rate of radial artery catheterization by palpation alone in pediatric patients was 18%-56% only [1, 5, 6]. With the rapid application of ultrasound visualization technology, the success rate of ultrasound-guided radial artery catheterization has been greatly ameliorated. However, the first success rate of radial artery catheterization for pediatric patients improved to 48%-83% only [7]. Failure of radial artery catheterization can lead to various complications such as vasospasm, local hematoma, and limb distal ischemia. Vasospasm of radial artery in turn can reduce the vascular diameter, change the vascular position, and further increase the difficulty of catheterization, which can even affect the safety of surgery [1, 8]. Therefore, searching an appropriate method to increase the diameter of radial artery and reduce the occurrence of vasospasm can improve the success rate of radial artery catheterization and facilitate the rapid recovery of pediatric patients, which is of great significance in the perioperative period.

Ultrasound-guided radial artery catheterization has been widely used in clinical practice in recent years and has been proved to improve the success rate, shorten the catheterization time, and reduce the radial complications effectively [9, 10]. This method is more suitable for patients with radial artery variation, low body weight, and low blood pressure in the elderly, especially those with pediatric radial artery hypoplasia and variability [11]. As a muscular artery with numerous elastic fibers, the radial artery is more sensitive to catecholamines in that it is mainly distributed by *α*-adrenergic receptors with few *β*-adrenergic receptors. In the process of catheterization, tension, pain, or mechanical stimulation on vessels may excite the sympathetic nervous system and then increase the catecholamine secretion, which can easily induce radial artery spasm [12]. As a short-acting, nonselective *α*-adrenergic receptor antagonist, phentolamine can competitively block the presynaptic *α*_2_ and postsynaptic *α*_1_ receptors, leading to the vasodilation and reduction of peripheral resistance. Since radial artery spasm is mainly mediated by *α*-adrenergic receptors, phentolamine can effectively reduce the occurrence of vasospasm [13]. Moreover, lidocaine is a local anesthesia drug with the superiority of faster transdermal absorption, longer action duration, and stable chemical properties. Subcutaneous injection of lidocaine can effectively block nerve conduction and relieve pain sensation caused by radial artery catheterization [14]. Thus, phentolamine accompanied by lidocaine subcutaneously may strongly reduce the secretion of vasoconstrictor substances, alleviate the stimulation of puncture pain, promote the dilation of radial smooth muscle, reduce peripheral vascular resistance and improve microcirculation. This study focused on the benefits of subcutaneous application of phentolamine alone or together with lidocaine on radial artery catheterization in pediatric patients, which may help to optimize the preoperative anesthesia process and reduce perioperative complications.

## 2. Materials and Methods

### 2.1. Subjects

This prospective, double-blinded, randomized controlled clinical trial was conducted to evaluate the superiority of phentolamine accompanied by lidocaine on radial artery catheterization in pediatric patients. We used the CONSORT 2010 checklist when writing our report [15]. This study was approved by the Committee on Ethics of Biomedicine of the 988th Hospital of Joint Logistic Support Force (20210017) and registered in the Chinese Clinical Trial Center (ChiCTR2100050672). Pediatric patients were well evaluated by the study group and acquired detailed information about the study. Pediatric patients meeting the inclusion criteria (less than 2 years old, American society of anesthesiologists (ASA) classification I-III, scheduled for elective surgery with invasive arterial blood pressure monitoring under general anesthesia) were enrolled in this study with written permission. Patients with peripheral vascular disease, severe atherosclerosis, hypersensitivity or contraindication to phentolamine or lidocaine, radial artery puncture recently, wound, infection or hematomas at the cannulation site, shock, or significant arrhythmia were excluded from the study.

### 2.2. Grouping

**Figure 1:**
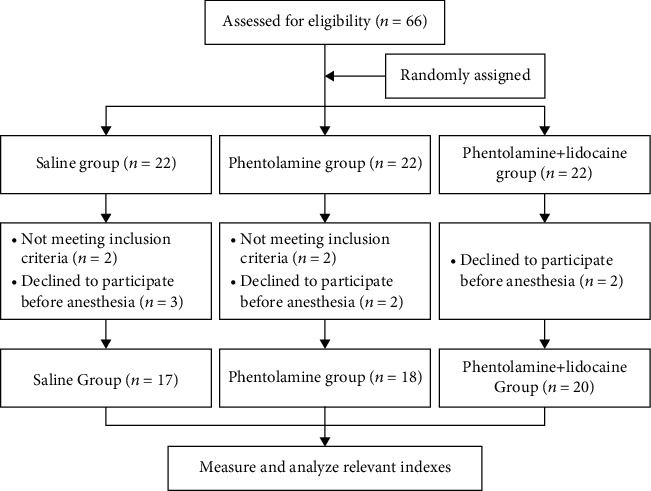
Flow diagram of grouping.

As the flow diagram shown in Figure 1, 66 pediatric patients were enrolled and analyzed in the saline group, phentolamine group, and phentolamine+lidocaine group. Baseline characteristics and surgical types of pediatric patients were collected. The double-blinded measures were conducted by sealing the number of patients in opaque envelopes that were opened by a well-trained nurse before induction of general anesthesia. Each envelope contained the group allocation with instructions for the subcutaneous drugs. Operators of ultrasound and radial artery cannulation were blinded to the patients' group allocation. Another anesthesiologist who measured the depth and diameter of the radial artery from the stored images was also blinded to the group allocation.

### 2.3. Procedural Protocol

The volume of 0.5 ml solution was determined to optimize the depth of the radial artery. A well-trained nurse prepared the syringe of drugs according to the group allocation. The saline group got 0.5 ml saline, the phentolamine group got 5 *μ*g/kg phentolamine in 0.5 ml solution, and the phentolamine+lidocaine group got 5 *μ*g/kg phentolamine and 4 mg/kg lidocaine in 0.5 ml solution. After general anesthesia, Allen's test was performed to ensure ulnar arterial patency and the right radial artery was chosen for routine catheterization. Noninvasive arterial blood pressure was measured every 2 minutes before radial artery cannulation on the right arm. After radial artery cannulation, invasive arterial blood pressure was monitored. Assessment of the radial artery and cannulation using ultrasonography was performed by pediatric anesthesiologists blind to group allocation. A short-axis view of the radial artery was obtained at the same location to assess the diameter and depth of radial artery before and after injection. In the three groups, 0.5 ml solution was subcutaneously injected around the radial artery over 10 seconds under ultrasound guidance to avoid intravascular injection. Arterial cannulation was performed using the long-axis view (in-plane) technique with a 24-gauge, 0.7 mm × 1.9 cm over-the-needle catheter. If cannulation was unsuccessful within the second attempt or within 10 minutes, the case was considered a failure on the chosen radial artery, and the contralateral side was used for cannulation without subcutaneous injection of drugs. The procedure time of arterial cannulation was defined as the time interval from the first skin puncture to the confirmation of the arterial waveform on the monitor. After the procedure, the diameter and depth of the radial artery, the first and second attempt success rate, and use of another artery were calculated, and the occurrence of complications were evaluated under ultrasonography. Vasospasm was defined as a more than 25% decrease in the artery diameter after cannulation; a more than 20% decrease in the mean blood pressure within 20 minutes after subcutaneous injection was regarded as hypotension [1].

### 2.4. Statistical Analysis

To study the benefit of phentolamine on radial artery cannulation with a statistical significance, we calculated the sample size and found that 60 patients were required (1 − *β* = 0.8, *α* = 0.05). All data was analyzed using GraphPad Prism 7 Software (San Diego, CA, USA). Quantitative data were expressed as mean ± standard deviation. One-way ANOVA were performed to compare data among all groups followed by Bonferroni's test; diameter and depth before and after injection were compared by paired *t*-test. Categorical data was expressed by percentage followed by the chi-square test. Kaplan–Meier analysis of the overall procedure time to successful cannulation of the chosen radial artery was performed, and the data were compared between the groups using the log-rank test. *P* < 0.05 was considered statistically significant.

## 3. Results

### 3.1. Baseline Characteristics of Pediatric Patients

The baseline characteristics of pediatric patients were shown in Table 1, and no significant differences were observed in age, sex, weight, and ASA classification among the three groups. Additionally, the vital signs in the three groups including systolic blood pressure (SBP), diastolic blood pressure (DBP), heart rate, and hemoglobin before anesthesia induction showed no significant differences. Also, cardiac surgery was the most commonly performed surgery in the three groups, followed by general surgery neurosurgery, while no significant differences were displayed in surgical types (Table 2).

### 3.2. Radial Artery Characteristics

As shown in Table 3, the internal diameter of the radial artery in the three groups showed no statistic differences before injection. After subcutaneous injection, the diameter in the phentolamine and phentolamine+lidocaine groups increased significantly compared with that in the saline group (*P* < 0.01). Moreover, the diameter also increased significantly after injection compared with that before injection both in the phentolamine and phentolamine+lidocaine groups (both *P* < 0.05). Moreover, the percentage changes of diameter before and after injection were higher in the phentolamine and phentolamine+lidocaine groups compared with that in the saline group (*P* < 0.01), indicating the vasodilator effect of phentolamine. However, there were no differences in the depth of the radial artery among the three groups throughout the procedure (Table 4).

### 3.3. Radial Artery Catheterization Data

**Figure 2:**
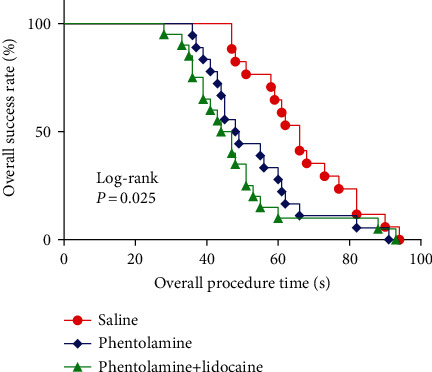
Kaplan–Meier analysis for the overall procedure time to successful cannulation.

In Table 5, the first-attempt success rates of radial artery cannulation were significantly higher (*P* < 0.05), while the procedure time to success within the first attempt, the procedure time to success within the second attempt and the overall procedure time of arterial cannulation were shorter (all *P* < 0.05) in the phentolamine and phentolamine+lidocaine groups than that in the saline group. The second-attempt success rate, use of another artery, and overall number of attempts were similar in the three groups. Kaplan–Meier analysis showed that the overall procedure time to successful cannulation was shorter in the phentolamine and phentolamine+lidocaine groups than that in the saline group (*P* < 0.05; Figure 2). Overall complications and vasospasm incidence were significantly lower in the phentolamine and phentolamine+lidocaine groups than that in the saline group (both *P* < 0.05). However, the incidence of hematoma, distal ischemia, and hypotension within 20 minutes after subcutaneous injection showed no significant differences in the three groups, and the phentolamine+lidocaine group showed a decreasing tendency in vasospasm and hematoma incidence (Table 6).

## 4. Discussion

Clinically, the major challenge to successful radial artery cannulation in pediatric patients is the small vascular diameter and sensibility to vasospasm. It is urgent to seek for new methods to increase the radial artery diameter and protect it from vasospasm and hematoma. The present study found that phentolamine accompanied by lidocaine subcutaneously before radial artery cannulation increased the diameter, which was essential for the increased first-attempt success rates and shorter procedure time to success within the first and second attempt. Moreover, phentolamine accompanied by lidocaine subcutaneously lowered the overall complications such as vasospasm and hematoma.

Radial artery has become the most commonly used site for arterial cannulation in monitoring invasive arterial pressure or coronary intervention for its superficial position and larger internal diameter. However, pediatric patients usually face greater difficulties in radial artery cannulation because of the low development, smaller internal diameter, and unexpectable variation of radial artery [16]. Studies have confirmed that many vasodilators could improve the success rate of radial artery cannulation and relieve vasospasm to a certain extent, such as nitrates, calcium channel antagonists, adrenergic receptor antagonists, or magnesium agents [17]. However, they also acquired various limitations and adverse reactions. Nitroglycerin can dilate most arteries and veins and relieve radial artery spasm effectively. Subcutaneous injection of nitroglycerin directly acts on the receptors of the radial artery to facilitate the release of endogenous nitric oxide and reduce intracellular calcium ions, followed by the dephosphorylation of the myosin light chain, resulting in the relaxing of vascular smooth muscle and dilating of radial artery vessels [18]. This effect can also increase the risk of headache and hypotension, which limits the application of nitroglycerin. Calcium channel antagonists such as verapamil, diltiazem, and nicardipine, can prevent calcium ions from flowing into cells by selectively acting on calcium channels on arterial smooth muscle. This effect can prevent arterial vasospasm, dilate vessels, increase arterial flow, and maintain the deformability of red blood cells [19]. Diltiazem gets a half-life of up to 3.5 hours, so it is not easy to control the drug metabolism by local injection [20]. Generally, calcium channel antagonists are used as adjunctive drugs in low dose as they get weaker effects on dilating arterial vessels and preventing spasm than nitrate and adrenergic receptor antagonists. In addition, as an antispasmodic drug, magnesium agents acquire the function of dilating blood vessels, lowering blood pressure, and improving local microcirculation, while they can also inhibit the nervous system and block the release of acetylcholine by nerve endings to ensure the relaxation of skeletal muscle. However, the spasmolysis efficacy of magnesium is still weak to dilate the diameter of radial artery for better cannulation without combining with other drugs [1, 21].

As a muscular artery with numerous elastic fibers, the radial artery is more sensitive to catecholamines. It is mainly distributed by *α*-adrenergic receptors with few *β*-adrenergic receptors. The occurrence of radial artery spasm involves various factors such as endothelin, thromboxane A2, or sympathetic molecules released by the endothelial cells. Moreover, the stretching caused by catheter during radial artery cannulation also induces spasm [22]. Meanwhile, mental tension, pain, or mechanical stimulation during the cannulation process can also excite the sympathetic nervous system to promote the secretion of catecholamines. The widely distributed *α*-adrenergic receptor mainly mediates the vasomotor process [23]. The present study tested the potential effects of phentolamine accompanied by lidocaine subcutaneously on the regulation of radial artery vasomotion. As a short-acting, nonselective *α*-adrenergic receptor antagonist, phentolamine can competitively block the presynaptic *α*_2_ and postsynaptic *α*_1_ receptors, leading to vasodilation and reduction of peripheral resistance. Since radial artery spasm is mainly mediated by *α*-adrenergic receptors, the use of phentolamine can effectively reduce the incidence of vasospasm. Subcutaneous injection of phentolamine also blocks the *α* receptor and dilates the radial artery in a relatively appropriate and effective way for its short half-life period, without a larger impact on the systemic circulation [24]. Studies have reported the low incidence of hypotension clinically by local application of phentolamine [25]. Phentolamine in combination with other drugs such as fasudil or diltiazem is also effective in preventing radial spasm in different mechanisms. Phentolamine pretreatment on the radial artery has been shown to be an effective way to prevent spasm in coronary artery grafts, but the effect on improving the success rate of radial artery cannulation has been reported rarely [26, 27]. Furthermore, effective analgesia before puncture can also significantly reduce vasospasm. Subcutaneous injection of lidocaine can block nerve impulses and the flow of ions across membranes to achieve the effect of anesthesia quickly [14]. The combined application of phentolamine and lidocaine can interact with multiple vasoconstriction factors to relieve the puncture stimulation and dilate the blood vessels, which may effectively relieve the spasm and improve the success rate of cannulation. The radial artery is susceptible to cannulation attempts, and the vasospasm or hematoma from failed attempts further decreases the internal diameter of artery, so the success at the first attempt is crucial during pediatric peripheral artery cannulation. Temporary vasospasm occurs in up to 57% of cases immediately after radial arterial cannulation, and sustained vasospasm occurs in 4% to 20% during trans-radial cardiac catheterization in adults [1, 28]. Vasospasm makes cannulation more difficult and decreases the success rate. A recent case report showed that the internal diameter decreased from 2.1 mm to 0.4 mm during radial artery spasm after failed cannulation in an 8-month-old infant [29]. This study also confirmed that phentolamine accompanied by lidocaine subcutaneously increased the diameter and first-attempt success rates and shortened the procedure time to success within the first and second attempts of arterial cannulation. Furthermore, the combined drugs subcutaneously lowered the overall complications such as vasospasm and hematoma.

## 5. Conclusions

In summary, phentolamine accompanied by lidocaine subcutaneously before radial artery cannulation increased the diameter and alleviated the vasospasm occurrence, which contributed to the increased success rates of radial artery catheterization under ultrasound guidance in pediatric patients.

## Figures and Tables

**Table 1:** Baseline characteristics of pediatric patients.

Characteristics	Saline group (*n* = 17)	Phentolamine group (*n* = 18)	Phentolamine+lidocaine group (*n* = 20)	*P* value
Age (months)	12.06 ± 4.68	10.28 ± 4.04	11.95 ± 4.05	0.469
Sex (male/female)	8/9	10/8	11/9	0.853
Weight (kg)	9.26 ± 1.63	9.01 ± 3.09	9.32 ± 3.03	0.932
ASA (%)				0.862
I	7 (41.18%)	6 (33.33%)	7 (35.00%)	
II	7 (41.18%)	9 (50.00%)	10 (50.00%)	
III	3 (17.65%)	3 (33.33%)	2 (10.00%)	
IV	0 (0%)	0 (0%)	1 (5.00%)	
V	0 (0%)	0 (0%)	0 (0%)	
SBP (mmHg)	83.53 ± 8.27	79.33 ± 9.37	82.15 ± 10.37	0.410
DBP (mmHg)	53.47 ± 6.79	52.06 ± 8.56	52.40 ± 6.78	0.842
Heart rate (min^−1^)	108.71 ± 14.41	106.28 ± 13.44	103.45 ± 12.36	0.494
Hemoglobin (g/dl)	11.44 ± 1.15	11.14 ± 1.56	11.08 ± 1.91	0.778

ASA: American Society of Anesthesiologists; SBP: systolic blood pressure; DBP: diastolic blood pressure.

**Table 2:** Surgical types.

Surgical types, *n* (%)	Saline group (*n* = 17)	Phentolamine group (*n* = 18)	Phentolamine+lidocaine group (*n* = 20)	*P* value
Neurosurgery	3 (17.65%)	2 (11.11%)	3 (15.00%)	0.997
Cardiac surgery	9 (52.94%)	9 (50.00%)	10 (50.00%)	
General surgery	3 (17.65%)	4 (22.22%)	3 (15.00%)	
Urinary surgery	1 (5.88%)	2 (11.11%)	2 (10.00%)	
Others	1 (5.88%)	1 (5.56%)	2 (10.00%)	

**Table 3:** Changes of radial artery diameter.

Variables	Saline group (*n* = 17)	Phentolamine group (*n* = 18)	Phentolamine+lidocaine group (*n* = 20)	*P* value
Diameter before injection (mm)	1.16 ± 0.11	1.16 ± 0.10	1.17 ± 0.10	0.906
Diameter after injection (mm)	1.13 ± 0.12	1.26 ± 0.15	1.32 ± 0.13	<0.01
*P* value	0.303	0.037	<0.01	
Diameter after catheterization (mm)	1.14 ± 0.11	1.28 ± 0.10	1.35 ± 0.09	<0.01
Percentage change of diameter before and after injection (%)	2.00 ± 0.13	10.00 ± 0.17	13.00 ± 0.17	<0.01

**Table 4:** Changes of radial artery depth.

Variables	Saline group (*n* = 17)	Phentolamine group (*n* = 18)	Phentolamine+lidocaine group (*n* = 20)	*P* value
Depth before injection (mm)	2.86 ± 0.11	2.83 ± 0.15	2.89 ± 0.17	0.561
Depth after injection (mm)	3.38 ± 0.16	3.29 ± 0.25	3.19 ± 0.32	0.091
*P* value	<0.01	<0.01	<0.01	
Depth after catheterization (mm)	3.42 ± 0.23	3.32 ± 0.21	3.22 ± 0.31	0.072

**Table 5:** Radial artery catheterization data.

Variables	Saline group (*n* = 17)	Phentolamine group (*n* = 18)	Phentolamine+lidocaine group (*n* = 20)	*P* value
First-attempt success rate (%)	10 (58.82%)	16 (88.89%)	18 (90.00%)	0.032
Procedure time to success within the first attempt (s)	56.50 ± 7.62	47.21 ± 7.43	42.71 ± 7.71	<0.01
Second-attempt success rate within 10 min (%)	14 (82.35%)	16 (88.89%)	18 (90.00%)	0.761
Procedure time to success within the second attempt (s)	61.79 ± 11.12	49.19 ± 9.59	43.67 ± 8.52	<0.01
Use of another artery (%)	3 (17.65%)	2 (11.11%)	2 (10.00%)	0.761
Overall procedure time of arterial cannulation (s)	66.53 ± 14.72	53.33 ± 15.13	48.35 ± 16.53	<0.01
Overall numbers of attempts	1.65 ± 0.93	1.33 ± 0.69	1.25 ± 0.64	0.262

**Table 6:** Complications of radial artery catheterization.

Variables	Saline group (*n* = 17)	Phentolamine group (*n* = 18)	Phentolamine+lidocaine group (*n* = 20)	*P* value
Vasospasm (%)	6 (35.29%)	2 (11.11%)	1 (5.00%)	0.035
Hematoma (%)	6 (35.29%)	3 (16.67%)	3 (15.00%)	0.268
Distal ischemia (%)	2 (11.76%)	1 (5.56%)	1 (5.00%)	0.691
Hypotension within 20 min after subcutaneous injection (%)	0 (0%)	0 (0%)	0 (0%)	
Overall complications	14	6	5	<0.01

## Data Availability

The datasets used and analyzed during the current study are available from the corresponding author on reasonable request.
